# Effects of High Temperature and High Pressure on the Photoluminescence of CdTe Quantum Dots: Implication for the High-Temperature Resistance Application of Nano-Stress Sensing Materials

**DOI:** 10.3390/ma18040746

**Published:** 2025-02-08

**Authors:** Jundiao Wang, Ke Bao, Yue Liu, Feihong Mao, Peirong Ren

**Affiliations:** 1 China North Vehicle Research Institute, Beijing 100072, China; bkbaoke@139.com (K.B.); 18510254023@163.com (Y.L.); fhmao0620@163.com (F.M.); 2 Chinese Scholartree Ridge State Key Laboratory, Beijing 100072, China; 3 School of Mechanical Engineering, Beijing Institute of Technology, Beijing 100081, China

**Keywords:** CdTe quantum dots, photoluminescence, high pressure, high temperature, stress sensing

## Abstract

Nano-sized quantum dots (QDs) have the potential for the application of stress sensing materials based on their pressure-sensitive photoluminescence (PL) properties, while the influence of a more realistic loading environment on the PL characteristics of QDs under a high-temperature environment remains to be further studied. Herein, we studied the PL response of CdTe QDs under repetitive loading–unloading conditions under high-temperature coupling to explore the stability of its high temperature stress sensing potential. The results show that the CdTe QDs with size of 3.2 nm can detect pressure in the range of 0–5.4 GPa, and the pressure sensitivity coefficient of PL emission peak energy ($E_{PL}$) is about 0.054 eV/GPa. Moreover, the relationship between $E_{PL}$ and pressure of CdTe QDs is not sensitive to high temperature and repeated loading, which meets the stability requirements of the sensing function required for stress sensing materials under high temperature. However, the disappearance of PL intensity caused by spontaneous growth as well as the ligand instability of QDs induced by high temperature/high pressure affects the availability of $E_{PL}$, which has a great influence on the application of CdTe QDs as high-temperature-resistant nano-stress sensing materials. The research provides the mechanical luminescence response mechanism of CdTe QDs under high-temperature/high-pressure coupling conditions, which provides experimental support for the design of high-temperature/high-pressure-resistant QD structures.

## 1. Introduction

The stress/strain sensing (SSs) device is an indispensable mechanical detection device in engineering. Traditional stress/strain (SS) gauges are mainly based on piezoresistive, capacitive and other principles, while new SSs devices involve fiber grating, wireless sensor and shape memory alloy, and so on [1,2,3]. Although the existing types of SSs devices are very diverse, it is still difficult to meet the needs for extreme application conditions. For example, for applications such as stress detection in military explosions, nuclear fusion and vehicles under extreme driving conditions, the load magnitude reaches the order of MPa or GPa, and the time resolution reaches the order of ns [4,5], while the existing SSs devices face difficulties in meeting the detection requirements. Besides this, for heterogeneous materials, the spatial resolution of SSs is usually required to reach the order of μm or nm. Moreover, in order to ensure that the structure of the measured material is not destroyed, wireless sensing is considered to be more convenient, which is difficult to achieve through the existing SSs materials [6]. The existing SSs materials or devices can hardly meet the detection requirements of high loads, high sensitivity and high precision, as well as being wireless, at the same time. Therefore, it is necessary to develop high-precision SSs materials through new materials.

For nano-semiconductor luminescent materials, such as QDs, nanowires, two-dimensional nano-materials, etc., in addition to the advantages of quantum computing, the pressure-induced PL spectral shift characteristics cause them to possess SSs potential [7,8,9,10,11]. For QDs, the zero-dimensional nanostructure gives them high-precision mechanical detection potential. It has been shown that CdTe QDs have many advantages as a very strong nano-semiconductor particle in the II-VI group [12]: high quantum yield, simple synthesis method [13], good chemical stability and size monodispersity [14]. Based on those advantages, the PL characteristics of CdTe QDs under different experimental conditions have been extensively studied and is expected to achieve sensing applications for different purposes such as SS, temperature, Ph value, etc. [9,15,16]. A large number of studies have confirmed that the PL spectra of CdTe QDs show a red shift with the increase in temperature, and show good temperature sensing stability in a certain temperature range [15,17,18,19]. Additionally, it is expected to achieve high-precision, high-sensitivity and wireless optically transmitted SSs by using the pressure-sensitive spectral shift characteristics of CdTe QDs. It has been confirmed that, at room temperature, the spectra of CdTe QDs with different sizes can shift to different degrees under hydrostatic pressure and impact pressure up to GPa [9,20,21]. Moreover, the sensitivity of wavelength to pressure reaches 1.8 nm/GPa, and the response time reaches the order of ns. In addition, the energy gap shift laws of CdTe QDs under different loading types of uniaxial compression, impact pressure and hydrostatic pressure were also compared and analyzed by first-principle simulation [22]. However, the studies on the SSs potential of CdTe QDs at present are mainly based on room-temperature environments, and the loading mode is mostly single loading–unloading. In fact, the application environment of SSs materials is often very complicated. The extreme environment requires that the SSs materials should not only be able to withstand high temperature but also have good mechanical stability under repeated loading and unloading conditions. Therefore, the current studies on the SSs potential of CdTe QDs are far from meeting the application requirements under the above extreme conditions.

In this paper, based on the practical application requirements of SSs materials, we explored the response of two important parameters, PL intensity and $E_{PL}$, with repetitive loading–unloading under high-temperature coupling conditions with a CdTe QD solution with a size of about 3.2 nm as the object. The results show that the relationship between the $E_{PL}$ of CdTe QDs and pressure represents a stable quadratic function, which is hardly affected by high temperatures and loading–unloading times. But unfortunately, high-temperature/high-pressure can aggravate the attenuation of PL intensity due to the instability of CdTe QDs ligands and the emergence of spontaneous growth, meaning that the $E_{PL}$, which can be used as a sensing index parameter, cannot be effectively obtained. The mechanism of mechanical-luminescence of CdTe QDs under complex high-temperature/high-pressure conditions is expounded based on experiment, which provides design inspiration for exploring more stable QDs for high-temperature/high-pressure applications.

## 2. Materials and Methods

**Figure 1:**
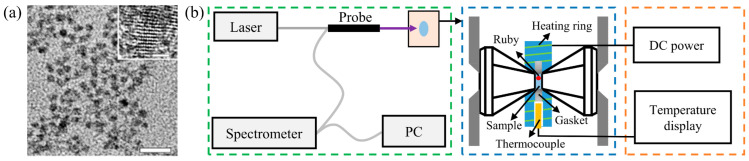
(**a**) Transmission electron microscope and high-resolution transmission electron microscope images of CdTe QDs. The scale bars in the transmission electron microscope and high-resolution transmission electron microscope correspond to 20 nm and 2 nm, respectively [23]. (**b**) Schematic of the composition of the experimental detection platform for the PL response of QDs under high -temperature/high-pressure.

The sample used in the experiment is a CdTe QD aqueous solution, which leads to the pressure type being close to hydrostatic pressure. For CdTe QDs samples, the shape is close to spherical and the size is close to 3.2 nm, as shown in Figure 1a [23]. The sample used in the experiment was provided by Huang et al. through the method of inhibiting Ostwald ripening by supplementing precursors before size defocusing in an aqueous synthesis system [23]. A more detailed preparation process, as well as the chemicals, can be accessed by consulting the research of Huang et al. We only briefly introduce the preparation method here: by using an injection pump device that can control the volume and rate of the precursor feed, the sample synthesis is carried out by programmed drop-by-drop addition of the precursor source, and finally, highly dispersed CdTe QDs are obtained. Among them, the precursor source is accurately the QDs with different sizes, that is, the QD precursors have grown into nanocrystals. And the characteristic of diameter distribution of the CdTe QDs can be confirmed through the paper of Huang et al.

The high-temperature/high-pressure loading device is built as shown in Figure 1b in order to study the effect of high-temperature/high-pressure environments on the PL properties of CdTe QDs. The QD solution and the pressure calibration material (ruby) are placed in the sample chamber composed of the gasket and the diamond anvil of the pressure loading unit. The external pressure forces the diamond pairs to approach each other, and the high-pressure environment of the QDs is realized with the narrowing of the sample cavity space. The PL spectra of QDs were collected by a spectrometer (Aurora 4000, with a detectable wavelength range of 200–1100 nm) when the laser (FC-D-405 nm) excites the QDs to analyze the sensitivity of PL to high-temperature/high-pressure. The change in temperature in the sample chamber is realized by connecting the DC (with a voltage range of 0–30 V) power supply and the heating ring outside the sample chamber with the thermocouple wire placed through the opening reserved by the heating ring to monitor the approximate temperature in the sample chamber. The responses of PL characteristics of CdTe QDs to repetitive loading–unloading at room temperature (about 298 K) and high temperature were studied by analyzing two important parameters of PL intensity and PL emission peak energy ($E_{PL}$) using the experimental equipment.

## 3. Results and Discussion

**Figure 2:**
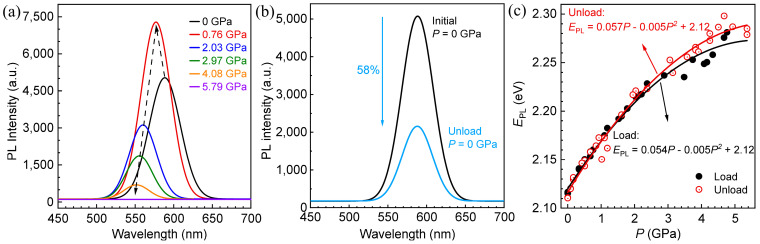
(**a**) Response of PL spectra of CdTe QDs with pressure. (**b**) Comparison of PL spectra of CdTe QDs before and after loading. (**c**) Response of $E_{PL}$ of CdTe QDs with pressure during loading–unloading.

The response of PL spectra of CdTe QDs to pressure during loading is shown in Figure 2a. Obviously, the PL intensity of CdTe QDs shows a non-monotonic function relationship with the pressure. When the pressure is lower than 0.76 GPa, the PL intensity shows a trend of increasing with loading pressure, while when the pressure reaches about 0.76 GPa, the PL intensity reaches its strongest point, which is about 1.5 times of the initial PL intensity. The reason for this phenomenon is mainly due to the fact that the low pressure increases the binding energy of the self-trapped exciton, and also causes the limited movement of the ligand, which leads to an effective reduction in the non-radiative recombination rate, and finally enhances the PL intensity of the QDs [24]. Then, as the pressure continues to increase, the PL intensity shows a continuous decay, consistent with experimental phenomena of other scholars [12,25]. This is mainly due to the distortion of the lattice of QDs and the QD–ligand bond under high pressure, resulting in an increase in the capture of photogenerated carriers by surface defects, thus showing a continuous decay of luminescence intensity [26]. When the pressure increases to about 5.79 GPa, the PL intensity basically disappears. For CdTe QDs with band-edge emission, previous studies attributed the quenching of pressure-induced PL to the occurrence of phase transition, that is, the lattice changed from zinc blende to rock salt, and the critical pressure for the disappearance of PL was close to the phase transition pressure [12,27]. The compared result of PL spectra of CdTe QDs before and after loading is shown in Figure 2b. It can be seen that the PL intensity after unloading represents a serious and irreversible attenuation compared with the initial intensity, and the attenuation degree is as high as about 58%, which is mainly due the fact that the pressure-induced distortion or fracture of the QD–ligand bond is difficult to recover [26].

The response relationship between $E_{PL}$ and pressure (P), as well as the fitting results during the loading–unloading process, is shown in Figure 2c. $E_{PL}$ and the emission wavelength (λ) satisfy the formula $E_{PL}=\frac{hc}{\lambda }$, where *h* is the Planck constant and *c* is the speed of light. During the loading process, the $E_{PL}$ increases with pressure. But when the pressure reaches about 5.40 GPa, $E_{PL}$ reaches the maximum value at about 2.28 eV, which can be observed due to the decay of the PL intensity. $E_{PL}$ is directly related to the energy gap of QDs, which mainly depends on the quantum confinement effect [28,29,30]. The dependence of $E_{PL}$ on pressure is mainly because of the decrease in the QD size and the lattice spacing caused by the increase of pressure, which enhances the quantum confinement effect and increases the energy gap, therefore causing $E_{PL}$ to increase with the loading pressure [30,31]. During the unloading process, $E_{PL}$ decreases with pressure, indicating that the decrease in pressure gradually restores the reduced lattice spacing. Moreover, when the pressure is reduced to zero, $E_{PL}$ is basically restored to the initial value. According to previous studies, $E_{PL}$ will decrease significantly if there is a fusion between QDs [32]. It can be inferred from the recovery of $E_{PL}$ after unloading that the QDs did not fuse. In order to describe the response law of $E_{PL}$ with pressure more accurately, empirical Formula (1) is used to fit the functional relationship between them [31,33].

(1) $$E_{PL}=\alpha P+\beta P^{2}+E_{0}$$

where *α* and *β* are the fitting parameters and $E_{0}$ is the fitting spectral peak energy at zero pressure. Function (1) with the above fitting parameters can be used as a guiding formula in pressure detection applications. The fitting results are listed in Figure 2c. By comparing the fitting results of loading and unloading, it is found that the deviation of fitted *α* obtained from the unloading process is only 5.6% compared with that from the loading process, while the fitted *β* and $E_{0}$ show good consistency. It can be seen that the $E_{PL}$ of CdTe QDs is more suitable as a pressure detection index compared to PL intensity.

**Figure 3:**
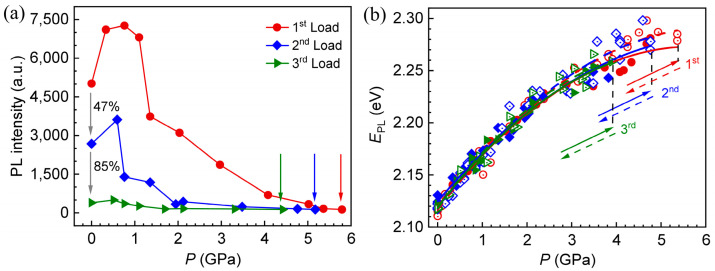
(**a**) Response of PL intensity of CdTe QDs with loading pressure during three continuous loading–unloading processes. The positions marked by the arrows represent luminescence quenching. (**b**) The evolution of $E_{PL}$ of CdTe QDs with pressure under three loading–unloading cycles (dots) and the corresponding fitting results (lines). The solid and hollow symbols represent loading and unloading results, respectively. The solid and dashed lines represent the fitting results of loading and unloading, respectively. Colors of red, blue and green represent the results of the first, second and third loading–unloading, respectively. The directions of the solid and dashed arrows represent the directions of loading and unloading, respectively.

Figure 3a records the change in PL intensity of CdTe QDs with loading pressure during three consecutive loading–unloading processes at room temperature. During this process, the response of PL intensity to pressure shows a consistent law, that is, the PL intensity increases with pressure under the low pressure range, while it shows the opposite trend under the high pressure range, which is consistent with the results shown in Figure 2a. However, the decay degree of the PL intensity and the critical pressure corresponding to the PL disappearance exhibit the following rules with the increase in the number of loading–unloading cycles:

First, with the increase in loading–unloading times, the PL intensity shows a continuous and irreversible decay, as shown in Figure 3a. The initial PL intensities of CdTe QDs were about 5014 a.u., 2680 a.u. and 402 a.u. during the three loading processes, showing an attenuation of 85% and 47%, respectively. According to the previous analysis, pressure-induced lattice distortion and QD–ligand bond distortion can lead to the capture of photogenerated carriers, which in turn attenuate the PL intensity of QDs. In addition, high pressure will not only cause the distortion of the QD–ligand bond, but also cause it to break, and the broken QD–ligand bond is difficult to recover [25]. Therefore, the PL intensity of CdTe QDs decreases continuously with the increase in loading–unloading times.

Second, the pressure values corresponding to the decay of the PL intensity of the QDs show a dependence on the number of loading–unloading cycles. As shown in Figure 3a, the pressure and PL intensity represented by the arrows correspond to baseline intensity and the pressure when the PL disappears, respectively. With the increase in loading times, the critical pressures corresponding to the disappearance of PL are about 5.79 GPa, 5.17 GPa and 4.42 GPa, respectively, showing a decreasing trend with the increase in loading–unloading times. The reason for this phenomenon is mainly due to the severe distortion and breakage of the QD–ligand bond caused by high pressure, which is difficult to recover, as described above. Furthermore, it has been confirmed that the connection ability between thioglycolic acid and CdTe QDs is weak, so high pressure can easily lead to the mutation of the QD–ligand bond, causing the removal of atoms on the surface of QDs, thereby inducing the decay of PL intensity [34,35].

The response of $E_{PL}$ of CdTe QDs with pressure during three loading–unloading cycles, as well as the fitting results obtained by formula (1), is shown in Figure 3b. All the fitting parameters are listed in Table 1. The response trajectories of $E_{PL}$ with pressure of three loading–unloading cycles are basically coincident, as exhibited in Figure 3b. Further, a clear law can be obtained by comparing the fitting parameters in Table 1. Specifically, compared with the parameter *α* obtained by the first loading fitting, the maximum deviation of the results obtained by the other fittings is about 7.4%, as listed in Table 1. For *β* and $E_{0}$, the fitting results of three loading–unloading cycles are basically the same. It can be concluded that the pressure sensitivity of $E_{PL}$ of CdTe QDs is basically independent of the number of loading–unloading cycles. It is worth mentioning that the maximum value of $E_{PL}$ and its corresponding pressure decrease with the increase in the cycle of loading–unloading, which is due to the dependence of PL intensity decay induced by high pressure on the number of loading–unloading cycles, as discussed above.

The actual working environments of SSs materials are usually at different temperatures. Whether the high-temperature/high-pressure coupling affects the pressure sensitivity of QD PL determines whether the performance of SSs materials based on CdTe QDs is stable. Based on this application demand, we continue to study the effect of high temperature on the pressure sensitivity of CdTe QDs. In the following, the effect of high temperature on the PL properties of CdTe QDs is studied first, and then the PL characteristic under high-temperature/high-pressure coupling is further studied and analyzed.

**Figure 4:**
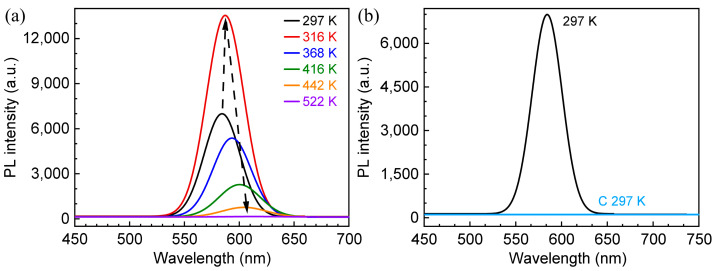
(**a**) Response of the PL spectra of CdTe QDs with increasing temperature. (**b**) Comparison of the PL spectra of CdTe QDs before and after the heating–cooling process.

The response of PL spectra of CdTe QDs with increasing temperature in the range of 297–522 K is shown in Figure 4a. With increasing temperature, the PL intensity of QDs shows an obvious non-monotonic function relationship with temperature. When the temperature is lower, the PL intensity gradually increases with temperature, and when the temperature reaches 316 K, the PL intensity reaches its strongest point, which is about 1.9 times the initial intensity. This phenomenon is similar to that of CdSe and CdTe/CdSe QDs in temperature experiments [17,36,37]. According to previous studies, high temperatures can induce a certain movement of the ligand on the surface of the QD, which makes the position of the atom connected to the ligand on the surface of the QD move to a certain extent, which leads to further relaxation of the surface of the quantum dot [37], thus enhancing the PL intensity because of the reduction in surface state defects of the QD. Then, as the temperature continues to rise, the PL intensity shows continuous attenuation, and the PL is close to disappearing until the temperature reaches 522 K. There are two reasons for the disappearance of PL caused by high temperatures. One is that the carriers at the deep traps induced by high temperatures are continuously emitted in a non-radiative manner, thereby resulting in the decay of PL intensity [17,38]. Another is that the spontaneous growth of QDs and the breaking of the QD–ligand bond can be caused by high temperatures, which leads to the increase in surface defects and accelerates the decay of luminescence intensity [39]. For the thioglycolic acid-modified CdTe QDs used in our experiment, the critical growth temperature is about 318 K [35]. The highest temperature of our experiment (522 K) is significantly higher than the critical growth temperature of CdTe QDs, so the spontaneous growth of CdTe QDs occurs. At the same time, when the temperature is close to or higher than the boiling point temperature of the ligand, the ligand will transform into a gaseous phase and be in a moving state, resulting in a completely “disconnected” state between the ligand and the QD, which not only leads to an increase in the surface defects of the QD, but also further increases the growth rate of the QD [39]. For the thioglycolic acid ligand used in CdTe QDs in our experiment, the boiling point is about 369 K, which is significantly lower than the highest temperature (522 K). Therefore, the gas phase state of the ligand plays a certain role in promoting the spontaneous growth of CdTe QDs.

Figure 4b presents the result of the comparison between the PL spectra of CdTe QDs after temperature cooling to room temperature and the initial spectra. Obviously, the PL of CdTe QDs is difficult to recover and almost disappears after high temperature, indicating that the high temperature (522 K) has an irreversible effect on the structure of CdTe QDs, which is due to the lack of ligands and the occurrence of Ostwald ripening, which will be discussed below.

In the high temperature experiment, at temperatures above the range of 297–522 K, the PL intensity of CdTe QDs was difficult to recover after cooling to room temperature, which makes it impossible to determine the temperature dependence of $E_{PL}$ during the cooling process. Therefore, in order to analyze the response of the $E_{PL}$ of CdTe quantum dots with temperature during the cooling process, the maximum temperature of the new experiment was reduced to 416 K, and the PL response of CdTe QDs during the heating–cooling process was studied again.

**Figure 5:**
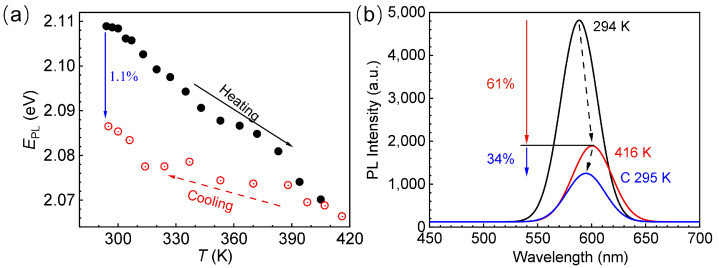
(**a**) Response of the $E_{PL}$ of CdTe QDs with temperature during the heating–cooling process. (**b**) Comparison of the PL spectra of CdTe QDs before and after the heating–cooling process.

The response of the $E_{PL}$ of CdTe QDs to temperature at the temperature range of 297–416 K is shown in Figure 5a. As the temperature increases, $E_{PL}$ decreases monotonously with the change in temperature, while as the temperature decreases, $E_{PL}$ increases in oscillation. Comparing the response trajectory of $E_{PL}$ with temperature during heating and cooling, it is found that the deviation between the two increases with the decrease in temperature, as illustrated in Figure 5a, which is consistent with other research results [35]. When the temperature is restored to room temperature, $E_{PL}$ decreases by about 1.1% compared with the initial value. The response of $E_{PL}$ during the cooling process is caused by the combined effect of spontaneous growth, temperature-dependent expansion of the lattice and the lattice–electron interaction. As mentioned above, high temperatures above the critical growth temperature of CdTe QDs can induce their spontaneous growth, resulting in a decrease in $E_{PL}$, and the process is irreversible. Therefore, when the temperature is reduced to room temperature, the deviation of the response trajectory of $E_{PL}$ with temperature is gradually increased compared with that during the heating process. However, under the action of high temperature, the temperature-dependent expansion of the CdTe QD lattice and the interaction between lattice and electron are better than its spontaneous growth effect, so $E_{PL}$ increases with the decrease in temperature.

As shown in Figure 5b, the PL spectra at different temperatures before and after the temperature rise are compared. Clearly, the PL intensity is always attenuated compared with the initial state, whether it is heating or cooling. When the temperature increases to 416 K, the PL intensity decreases by about 61% compared with the initial state (294 K), and when the temperature is reduced to 295 K, the PL intensity continues to decay on the basis of 416 K, and the attenuation degree is about 34%, which is caused by the lack of ligands due to the high temperature, as mentioned above. However, the fact that the PL of CdTe QDs is difficult to recover when the temperature is cooled from 522 K to room temperature, as shown in Figure 4b, mainly depends on the occurrence of Ostwald ripening in addition to the induction of ligand loss [39]. Ostwald ripening refers to the dissolution of small-sized QDs and regrowth to larger-sized QDs, thereby accelerating the growth of QDs. However, the grown QDs cannot obtain sufficient and stable ligands for passivation, which is accompanied by a large number of surface defects. Therefore, when the temperature is cooled from 522 K, the PL intensity of CdTe QDs still cannot be restored.

**Figure 6:**
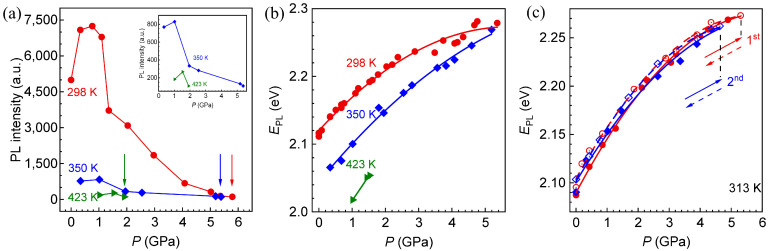
(**a**) Response of PL intensity of CdTe QDs with pressure at different temperatures. The positions marked by arrows represent luminescence quenching. (**b**) Response of $E_{PL}$ of CdTe QDs with pressure at different temperatures. (**c**) Response of $E_{PL}$ of CdTe QDs with pressure under repetitive loading–unloading and the fitting results (*T* = 313 K).

The response of the PL intensity of CdTe QDs to pressure at different high temperatures is shown in Figure 6a. For different high-temperature environments, the response of the PL intensity to pressure shows a non-monotonic change: under lower pressure, the PL intensity increases with pressure. Subsequently, with continued loading, the PL intensity shows a decreasing trend, which is consistent with the law at room temperature in Figure 2a. For the temperatures of 350 K and 423 K, the detailed response law of PL intensity with pressure can be observed from the illustration. But the initial pressure environment of the QD solution and the corresponding relationship between PL intensity and pressure show temperature dependence:

Firstly, the initial environmental pressure of the CdTe QD solution exhibits temperature dependence. When the corresponding temperatures are 298 K, 350 K and 423 K, the initial pressure environments are 0 GPa, 0.34 GPa and 1.02 GPa, respectively, increasing with temperature, which is due to the expansion of the matrix solution caused by high temperature in the sealed environment, resulting in pre-pressure.

Secondly, the pressure required for the PL intensity to reach the maximum value exhibits temperature dependence. The corresponding temperatures are 298 K, 350 K and 423 K, and the pressures required to reach the highest PL intensity are about 0.76 GPa, 0.67 GPa and 0.51 GPa, respectively, showing a decreasing trend with the increase in temperature. It can be concluded that with the increase in temperature, the decay of PL intensity occurs at lower pressure, which is caused by different phase transitions of ligands induced by high temperature [39]. The melting point and boiling point of thioglycolic acid are 257 K and 369 K, respectively. For 298 K and 350 K in Figure 6a, the thioglycolic acid is in the liquid phase, showing a stable movement on the surface of the QD, and the connection with the atoms on the surface of the QD is between the ‘on’ and ‘off’ states, while for 423 K, thioglycolic acid is in the gaseous phase, showing a complete motion state, and the connection with the surface of QD is completely disconnected. Therefore, with the increase in temperature, the lack of ligands on the surface of QDs becomes more and more serious, so that the lower pressure can induce the distortion of the lattice and QD–ligand bond of QDs and the capture of photogenerated carriers [26], thus making the PL intensity of QDs more likely to decay.

Thirdly, the pressure corresponding to the disappearance of PL exhibits temperature dependence. The pressure and PL intensity, represented by the arrows, correspond to baseline intensity and the pressure when the PL disappears, respectively, as shown in Figure 6a. At 298 K, 350 K and 423 K, the loading pressures required for the disappearance of PL are about 5.79 GPa, 5.04 GPa and 0.89 GPa, respectively, showing that the former two are close to each other, while the third is greatly reduced. For 298 K and 350 K, according to the previous analysis, the ligand is in the liquid phase; even if it shows a certain movement on the surface of the QD, it can still be regarded as quasi-static [39]. Moreover, the loading pressure can greatly limit the movement of the ligand, resulting in a small difference in the effect of the ligand on the surface of the QD at 350 K compared with 298 K. However, for 423 K, the ligand is in a state of complete motion, and the connection with the surface of the QD is basically disconnected, accompanied by the spontaneous growth of the QD. Therefore, lower pressure can cause a significant increase in the surface defects of the QDs and cause the PL to disappear. It can be seen that when the temperature is higher than the boiling point of the ligand and the temperature threshold of the spontaneous growth of QDs, the high-temperature/high-pressure coupling will aggravate the disappearance of PL.

The relationship between $E_{PL}$ and pressure at different temperatures and the fitting results of formula (1) are illustrated in Figure 6b. The fitting parameters are listed in Table 2. Firstly, the initial $E_{PL}$ in different loading trajectories decreases with the increase in temperature, which is consistent with the results of Figure 5a. Secondly, the response trajectory of $E_{PL}$ with pressure is shifted to the right with the increase in temperature due to the generation of pre-pressure. Finally, the maximum deviation of *α* at different temperatures is found to be about 9.2% through the comparison of the data in Table 2, which is close to the maximum relative deviation (about 8%) in Figure 3b. It can be seen that *α* shows good consistency under different high-temperature conditions, indicating that the lattice expansion and lattice–electron interaction induced by high temperature have little effect on the pressure sensitivity of CdTe QDs. However, there is a big difference in *β* at different temperatures. For 298 K and 350 K, the direct reason for the difference in the obtained *β* is the fact that when the pressure is higher than 3.5 GPa, $E_{PL}$-P trajectories of the two groups are gradually approaching. For 423 K, the loading pressure required for the disappearance of PL is greatly reduced, resulting in a significant reduction in the fitting data and a large *β* deviation. It can be seen that when the temperature is lower than the boiling point of the ligand and the growth temperature of the QDs, and the pressure is lower than 3.5 GPa, the high-temperature/high-pressure coupling has little effect on the pressure sensitivity of CdTe QDs.

According to the influence of temperature on the pressure dependence of PL intensity and $E_{PL}$ in Figure 6a,b, it can be seen that the temperature below 350 K is more conducive to the development of loading–unloading cycle experimental research. Therefore, we choose to carry out repetitive loading–unloading experiments on CdTe QDs in a constant temperature environment of 313 K to explore the stability of the response law of PL characteristics with pressure under repeated loading–unloading conditions in a high-temperature coupling environment. In addition, the repetitive pressure experiment of CdTe QDs at room temperature in Figure 3 proves that the response of $E_{PL}$ with pressure is basically independent of the number of loading–unloading cycles, so the number of cycles at 313 K is set to twice.

The response of the $E_{PL}$ of CdTe QDs to the loading–unloading cyclic pressure at 313 K, as well as the fitting result of Formula (1), is shown in Figure 6c. The fitting parameters are listed in Table 3. Firstly, the pre-pressure caused by the high temperature of 313 K is almost zero, as illustrated in Figure 6c. Secondly, the trajectories of $E_{PL}$ of different loading–unloading cycles with pressure are basically the same, and the fitting results are very close (Table 3), which is consistent with that at room temperature in Figure 3. Finally, the maximum $E_{PL}$ can be detected by spectrometer, and its corresponding pressure shows the dependence of the number of loading–unloading cycles, which is consistent with that at room temperature in Figure 3. It can be seen that the high temperature within a certain range, i.e., when the temperature is lower than the boiling point of the ligand, as well as the growth temperature of the QDs, basically does not affect the relationship between the $E_{PL}$ and pressure of CdTe QDs under repetitive loading–unloading.

## 4. Conclusions

In summary, high temperature and high pressure are used as experimental variables to study the PL response of CdTe QDs when they act separately and couple with each other. The effects of repetitive pressure on PL characteristics of CdTe QDs with high-temperature coupling are further investigated. It can be judged from the following aspects that CdTe QDs have the application potential of SSs materials with high temperature and high pressure resistance. Firstly, for the thioglycolic acid-modified CdTe QDs with a size of about 3.2 nm, the maximum detectable pressure is about 5.40 GPa based on $E_{PL}$, showing high pressure resistance. Secondly, the relationship between $E_{PL}$ and pressure satisfies a quadratic function which is not affected by loading times and high temperature, showing good stability, and can be used as a guiding relationship for pressure sensing. Thirdly, the dependence of the $E_{PL}$ of CdTe QDs on the number of loading–unloading cycles and high temperature is mainly due to the decay of the PL intensity caused by the instability of the ligand and the spontaneous growth of QDs. Therefore, for the temperature of the application environment below that of the spontaneous growth of CdTe QDs, selecting a more stable ligand or shell for QD surface modification can improve the stability of PL intensity, which is helpful for the realization of SSs application with high temperature resistance for CdTe QDs. This research can provide a reference for revealing the mechanical-luminescence mechanism of CdTe QDs under high-temperature/high-pressure coupling and inspiration for the improved design of QDs used for SSs materials.

## Figures and Tables

**Table 1:** Fitting parameters in Figure 3b.

Cycle		*α* (eV/GPa)	*β* (eV/GPa^2^)	$E_{0}$ (eV)
1st	Load	0.054	−0.005	2.12
	Unload	0.057 (5.6%)	−0.005	2.12
2nd	Load	0.050 (7.4%)	−0.004	2.12
	Unload	0.057 (5.6%)	−0.005	2.12
3rd	Load	0.050 (7.4%)	−0.004	2.12
	Unload	0.057 (5.6%)	−0.005	2.12

**Table 2:** Fitting parameters in Figure 6b.

*T* (K)	*α* (eV/GPa)	*β* (eV/GPa^2^)	$E_{0}$ (eV)
298	0.054	−0.005	2.12
350	0.059 (9.2%)	−0.003	2.05
423	0.056 (3.7%)	0.007	1.95

**Table 3:** Fitting parameters in Figure 6c.

Cycle		*α* (eV/GPa)	*β* (eV/GPa^2^)	$E_{0}$ (eV)
1st	Load	0.060	−0.005	2.09
	Unload	0.058 (3.3%)	−0.005	2.10
2nd	Load	0.053 (11.7%)	−0.004	2.10
	Unload	0.059 (1.7%)	−0.005	2.10

## Data Availability

Data are contained within the article.

## References

[1] Farooq M., Iqbal T., Vazquez P., Farid N., Thampi S., Wijns W., Shahzad A. (2020). Thin-Film Flexible Wireless Pressure Sensor for Continuous Pressure Monitoring in Medical Applications. Sensors.

[2] Schenato L., Galtarossa A., Pasuto A., Palmieri L. (2020). Distributed optical fiber pressure sensors. Opt. Fiber Technol..

[3] Yan W., Wang C.H., Zhang X.P., Mai Y.-W. (2003). Theoretical modelling of the effect of plasticity on reverse transformation in superelastic shape memory alloys. Mater. Sci. Eng. A.

[4] Kato Y., Murata K., Kubota S. (2023). Measurements of Shock and Detonation Phenomena. Detonation Phenomena of Condensed Explosives.

[5] Zhang G., Zhao Y., Zhao Y., Wang X., Wei X., Ren W., Li H., Zhao Y. (2018). A Manganin Thin Film Ultra-High Pressure Sensor for Microscale Detonation Pressure Measurement. Sensors.

[6] Gray G.T. (2012). High-strain-rate deformation: Mechanical behavior and deformation substructures induced. Annu. Rev. Mater. Res..

[7] Das P., Ganguly S., Rosenkranz A., Wang B., Yu J., Srinivasan S., Rajabzadeh A.R. (2023). MXene/0D nanocomposite architectures: Design, properties and emerging applications. Mater. Today Nano.

[8] Di Carlo V., Prete P., Dubrovskii V.G., Berdnikov Y., Lovergine N. (2017). CdTe Nanowires by Au-Catalyzed Metalorganic Vapor Phase Epitaxy. Nano Lett..

[9] Kang Z., Banishev A.A., Lee G., Scripka D.A., Breidenich J., Xiao P., Christensen J., Zhou M., Summers C.J., Dlott D.D. (2016). Exploration of CdTe quantum dots as mesoscale pressure sensors via time-resolved shock-compression photoluminescent emission spectroscopy. J. Appl. Phys..

[10] Shahi K., Singh R., Singh A.K., Aleksandrova M., Khenata R. (2018). CdTe quantum-dot-modified ZnO nanowire heterostructure. Appl. Phys. A.

[11] van Riggelen-Doelman F., Wang C.-A., de Snoo S.L., Lawrie W.I., Hendrickx N.W., Rimbach-Russ M., Sammak A., Scappucci G., Déprez C., Veldhorst M. (2024). Coherent spin qubit shuttling through germanium quantum dots. Nat. Commun..

[12] Lin Y.C. (2013). Water-soluble CdTe nanocrystals under high pressure. Quantum Dots and Nanostructures: Synthesis, Characterization, and Modeling XII.

[13] He Y., Sai L.-M., Lu H.-T., Hu M., Lai W.-Y., Fan Q.-L., Wang L.-H., Huang W. (2007). Microwave-assisted synthesis of water-dispersed CdTe nanocrystals with high luminescent efficiency and narrow size distribution. Chem. Mater..

[14] Wang Q., Kuo Y., Wang Y., Shin G., Ruengruglikit C., Huang Q. (2006). Luminescent properties of water-soluble denatured bovine serum albumin-coated CdTe quantum dots. J. Phys. Chem. B.

[15] Liang R., Tian R., Shi W., Liu Z., Yan D., Wei M., Evans D.G., Duan X. (2013). A temperature sensor based on CdTe quantum dots–layered double hydroxide ultrathin films via layer-by-layer assembly. Chem. Commun..

[16] Zhang C., Xu J., Zhang S., Ji X., He Z. (2012). One-Pot Synthesized DNA-CdTe Quantum Dots Applied in a Biosensor for the Detection of Sequence-Specific Oligonucleotides. Chem.-A Eur. J..

[17] Chin P.T.K., de Mello Donegá C., van Bavel S.S., Meskers S.C.J., Sommerdijk N.A.J.M., Janssen R.A.J. (2007). Highly Luminescent CdTe/CdSe Colloidal Heteronanocrystals with Temperature-Dependent Emission Color. J. Am. Chem. Soc..

[18] Chon B., Bang J., Park J., Jeong C., Choi J.H., Lee J.-B., Joo T., Kim S. (2011). Unique Temperature Dependence and Blinking Behavior of CdTe/CdSe (Core/Shell) Type-II Quantum Dots. J. Phys. Chem. C.

[19] Zhang P., Pan A., Yan K., Sun J. (2023). High stability temperature sensors by CdTe quantum dots encapsulated in SiO2/PVA hybrids for bearing rotating elements. Mater. Today Commun..

[20] Wu F., Zaug J.M., Young C.E., Zhang J.Z. (2008). Pressure-induced phase transition in thiol-capped CdTe nanoparticles. J. Nanosci. Nanotechnol..

[21] Xiao P., Ke F., Bai Y., Zhou M. (2017). Deformation-induced blueshift in emission spectrum of CdTe quantum dot composites. Compos. Part B Eng..

[22] Wang J., Shi R., Xiao P. (2023). The effect of loading modes on the strain-dependent energy gap of CdTe quantum dots: A first-principles study. Comput. Mater. Sci..

[23] Huang X., Jing L., Kershaw S.V., Wei X., Ning H., Sun X., Rogach A.L., Gao M. (2018). Narrowing the photoluminescence of aqueous CdTe quantum dots via ostwald ripening suppression realized by programmed dropwise precursor addition. J. Phys. Chem. C.

[24] Wang Y., Guo S., Luo H., Zhou C., Lin H., Ma X., Hu Q., Du M.-h., Ma B., Yang W. (2020). Reaching 90% photoluminescence quantum yield in one-dimensional metal halide C4N2H14PbBr4 by pressure-suppressed nonradiative loss. J. Am. Chem. Soc..

[25] Liu H., Yang X., Wang K., Wang Y., Wu M., Zuo X., Yang W., Zou B. (2020). Pressure-induced multidimensional assembly and sintering of CuInS2 nanoparticles into lamellar nanosheets with band gap narrowing. ACS Appl. Nano Mater..

[26] Zhou B., Xiao G., Yang X., Li Q., Wang K., Wang Y. (2015). Pressure-dependent optical behaviors of colloidal CdSe nanoplatelets. Nanoscale.

[27] Freire P., Silva M.A., Reynoso V., Vaz A., Lemos V. (1997). Pressure Raman scattering of CdTe quantum dots. Phys. Rev. B.

[28] Cingolani R., Di Dio M., Lomascolo M., Rinaldi R., Prete P., Vasanelli L., Vanzetti L., Bassani F., Bonanni A., Sorba L. (1994). Photocurrent spectroscopy of Zn 1− x Cd x Se/ZnSe quantum wells in p-i-n heterostructures. Phys. Rev. B.

[29] Sapra S., Sarma D. (2004). Evolution of the electronic structure with size in II-VI semiconductor nanocrystals. Phys. Rev. B.

[30] Zhao H., Yin H., Liu X., Li H., Shi Y., Liu C., Jin M., Gao J., Luo Y., Ding D. (2019). Pressure-induced tunable electron transfer and Auger recombination rates in CdSe/ZnS quantum dot–anthraquinone complexes. J. Phys. Chem. Lett..

[31] Yuan C.-T., Lin Y., Chen Y.-N., Chiu Q., Chou W.-C., Chuu D., Chang W., Lin H., Ruaan R., Lin C. (2007). Studies on the electronic and vibrational states of colloidal CdSe/ZnS quantum dots under high pressures. Nanotechnology.

[32] Cui J., Panfil Y.E., Koley S., Shamalia D., Waiskopf N., Remennik S., Popov I., Oded M., Banin U. (2019). Colloidal quantum dot molecules manifesting quantum coupling at room temperature. Nat. Commun..

[33] Lin Y.-C., Chou W.-C., Susha A.S., Kershaw S.V., Rogach A.L. (2013). Photoluminescence and time-resolved carrier dynamics in thiol-capped CdTe nanocrystals under high pressure. Nanoscale.

[34] Zhou D., Lin M., Liu X., Li J., Chen Z., Yao D., Sun H., Zhang H., Yang B. (2013). Conducting the temperature-dependent conformational change of macrocyclic compounds to the lattice dilation of quantum dots for achieving an ultrasensitive nanothermometer. ACS Nano.

[35] Zhou D., Zhang H. (2013). Critical Growth Temperature of Aqueous CdTe Quantum Dots is Non-negligible for Their Application as Nanothermometers. Small.

[36] Wuister S.F., de Mello Donegá C., Meijerink A. (2004). Luminescence temperature antiquenching of water-soluble CdTe quantum dots: Role of the solvent. J. Am. Chem. Soc..

[37] Wuister S.F., Van Houselt A., de Mello Donegá C., Vanmaekelbergh D., Meijerink A. (2004). Temperature antiquenching of the luminescence from capped CdSe quantum dots. Angew. Chem. Int. Ed..

[38] Liu T.-C., Huang Z.-L., Wang H.-Q., Wang J.-H., Li X.-Q., Zhao Y.-D., Luo Q.-M. (2006). Temperature-dependent photoluminescence of water-soluble quantum dots for a bioprobe. Anal. Chim. Acta.

[39] Pradhan N., Reifsnyder D., Xie R., Aldana J., Peng X. (2007). Surface ligand dynamics in growth of nanocrystals. J. Am. Chem. Soc..

